# Evaluation of the Medical Resource Allocation: Evidence from China

**DOI:** 10.3390/healthcare11060829

**Published:** 2023-03-11

**Authors:** Yueming Xi, Ye Ding, Yenuan Cheng, Junjie Zhao, Mengqiu Zhou, Shangren Qin

**Affiliations:** 1 School of Public Health, Hangzhou Normal University, Hangzhou 311121, China; 2 School of Public Health, Hangzhou Medical College, Hangzhou 311399, China

**Keywords:** medical resource allocation, China, evaluation, satisfaction, cross-sectional study

## Abstract

Medical resource allocation is an increasingly crucial issue. It is vital to understand residents’ (people living in the local area) evaluation of it. This study explores residents’ evaluation of medical resource allocation and its determinants with the dimensions of medical resource adequacy, balance, publicness, and accessibility. We used data from the China General Social Survey (CGSS). Binary logistic regression models were constructed from the four dimensions separately, and we compared the differences among them. The study’s results showed that accessibility and publicness are the highest and lowest dimensions of residents’ evaluation, respectively. The high evaluation of social equity may be a positive contributor to a higher evaluation of publicness and accessibility. The central region residents had the lowest evaluation of adequacy (OR = 0.697, *p* = 0.010) and balance (OR = 0.642, *p* = 0.008). To sum up, perceived social equity, social trust, and social class are crucial factors. Based on the results, the government should strengthen the supervision of the medical market, increase financial support for the health field in the central region, and establish a multi-level medical security system that may help optimize the allocation.

## 1. Introduction

The allocation of medical resources is a central issue in health policy making [1]. The equitable allocation of medical resources is one of the main objectives of the government’s interventions in the health services market [2]. The allocation of medical resources is not simply a spatial distribution, but pays attention to whether the structure of medical resource allocation is reasonable and equitable [3], and its goal is to maximize the efficiency and effectiveness of allocation [2].

The allocation of medical resources is an increasingly critical issue worldwide [4], as it is in China. Since the reform and opening up in 1978, people’s living standards had improved due to the rapid development of China’s economy, science, and technology. Meanwhile, people’s demand for a better life was growing and their attention to their own health was also increasing unprecedentedly [5]. However, the problem of insufficient and imbalanced allocation of medical resources gradually emerged. In addition, the health care system trended toward market-oriented during its development, which weakened the publicness and the accessibility of medical services, and the rapid rise of medical costs made medical expenses unaffordable to residents. To realize equitable and accessible allocation of medical resources, the Chinese government took many measures. In 2009, the Chinese government initiated a plan to launch a new round of reforms of the health care system, and pledged to provide basic health services, covering both urban and rural residents equally, by 2020 [6]. To this end, the Chinese government increased investment in medical resources, especially increased fiscal investment to the central and western regions, expanded the coverage of basic medical insurance, improved primary health services, promoted the construction of a hierarchical diagnosis and treatment system, and narrowed the gap in medical resource allocation between developed and less developed regions. However, the contradictions between health needs, scarce medical resources, and the unequal allocation of medical resources have always existed. To allocate medical resources equitably and reasonably, studying the demand side of medical resources, namely residents’ evaluation of medical resource allocation, is crucial.

The equitable allocation of medical resources is a central objective pursued by the health care systems of many countries [7]. Previous studies on the equity of medical resource allocation used data from various health statistics yearbooks to evaluate the equity of allocation, for example, by calculating the Gini coefficient to evaluate the equality based on geographic area [8]. Another group of researchers studied residents’ evaluation of the overall equity of the health care system [9]. However, only a few researchers have taken residents’ evaluation of the equity of the medical resource allocation as their research content. Existing research showed that residents’ evaluation of the equality of medical resource allocation was influenced by socioeconomic factors, such as income, employment, and health investment [10]. In the United States, the disparities in residents’ perceived equity of medical resource allocation were closely related to income inequality. The large proportion of out-of-pocket medical expenditures and low medical insurance coverage in the United States may leave the health needs of low-income people and disadvantaged groups unmet [11]. In China, relevant studies showed that hukou, perceived social status, and medical insurance participation influenced residents’ perceived equity of health services [12]. Although the health care systems vary from country to country, the goal of health care reform is to promote equity in health care systems. However, equity in the health care system is not a simple concept; it consists of a combination of concepts such as accessibility of health care resources [13], affordability of health care expenditures, reduction in health disparities [14], and balanced allocation of medical resources [15].

Understanding residents’ perceived accessibility to medical resource allocation is essential to promote patient-centered health services, which is related to factors such as the type of medical insurance plan, costs of medical insurance premiums, advanced age, marital status, and perceived health status [16]. The analysis of the elderly population’s accessibility of medical resources showed that income and perceived accessibilities were related to individual’s socioeconomic status [17]. Not only that, but employment, income, and medical insurance participation all had an impact on the evaluation of accessibility [18]. Balanced allocation of medical resources is the basis for sustainable development of health undertaking [19]. In China, the imbalance of economic development levels between regions and between urban and rural areas still exists. Regional fiscal differences may lead to differences in the supply of public services between regions [20]. The allocation of medical resources is affected by differences in regional economic levels, and the government’s investment in medical resources continues to increase in developed regions, while it is insufficient in less developed regions, thus leading to the problem of unequal allocation of medical resources in different regions. However, most of the studies investigated the balance of medical resource allocation through the objective data from statistical yearbooks [21], but few analyzed residents’ evaluation of the balance of medical resource allocation. 

The research on medical resource allocation has shown a diversified trend, and the vital role of marketization mechanisms on resource allocation has become one of the important contents of the study. The implementation of medical marketization transforms the price of medical services from government regulation to market regulation, and enables medical resources to be allocated efficiently [22]. In China, the publicness and public welfare are important characteristics of medical and health services [23]. “Publicness” means every resident has access to medical resources and can afford medical expenses. However, excessive medical marketization will lead to inequity of medical services. In the case of medical marketization, patients’ ability to pay will be an important factor in determining hospital income, while the difference in payment ability will lead to inequity of medical services, and poor people will have difficulty in obtaining high-quality medical services. Therefore, in order to make better suggestions on health reform, it is necessary to understand residents’ views on the publicness and marketization of medical resource allocation.

In summary, the existing studies mainly focused on the allocation of medical resources, residents’ overall satisfaction with medical resource allocation, or residents’ evaluation of medical resources allocation and the influencing factors of a single dimension, such as equity or accessibility. However, these studies did not refine the evaluation dimension of satisfaction and conduct comparative studies from multiple dimensions such as adequacy, balanced distribution, publicness, and accessibility of medical resources.

Therefore, using data from the 2013 Chinese General Social Survey (CGSS2013), this study explores Chinese residents’ evaluation of medical resource allocation and the influencing factors in four dimensions: adequacy, balance, publicness, and accessibility of medical resource allocation. Moreover, it conducts a comparative study to provide a reference for policy formulation on medical resource allocation in China. 

## 2. Materials and Methods

### 2.1. Data and Research Design

Data in this study were derived from the 2013 China General Social Survey (CGSS) to classify residents’ evaluation of medical resource allocation into four dimensions: adequacy, balance, publicness, and accessibility.

Part of the independent variables in this study were selected from variables in Anderson’s behavioral model, which is a mainstream model applicable to health service research [24]. This study chose independent variables in the dimensions of personal characteristics in the model: sex, age, marital status, job, income, perceived health status, and medical insurance status. In addition, hukou, perceived social trust, perceived social equity, perceived social class, and economic zone were included in the independent variables. Hukou is a mandatory household registration system in China. Each resident can only register a unique residence address, and hukou has two types: urban hukou and rural hukou. The data were analyzed by chi-square test and binary logistic regression model to explore residents’ evaluation of the four dimensions of medical resource allocation and to analyze their influencing factors.

Data in this study were derived from the 2013 China General Social Survey (CGSS), which began in 2003 and was implemented by the National Survey Research Center at Renmin University of China (NSRC). The CGSS collected comprehensive data at multiple levels, including society, community, family, and individual, to systematically monitor residents’ behavior and attitudes toward radical social change.

The CGSS2013 used a stratified three-stage sampling method to survey residents (including urban and rural hukou) in 31 provinces in mainland China. The survey was stratified into two tiers: the first tier, the mandatory tier, was the residents in municipal districts of the more developed megalopolis, including Shanghai, Beijing, Tianjin, Shenzhen, and Guangzhou. The second tier, the sampling tier, was for all residents except the mandatory tier.

The CGSS2013 data used in this study covered a total of 12,000 households and obtained a valid sample of 11,438. CGSS2013 data contain the questions on residents’ evaluation of medical resource allocation needed for this study. Although CGSS2015 and 2017 have been published, they do not cover the questions required for this study, so it is still valuable to choose CGSS2013.

The CGSS data are open and could be downloaded at the official website (http://www.cnsda.org/index.php?r=projects/view&id=93281139 (accessed on 15 June 2022)) after registration and review.

### 2.2. Variables and Measurements

***Evaluation of Medical Resource Allocation***. According to the CGSS questionnaire setting, the dependent variable, Evaluation of Medical Resource Allocation, is divided into four dimensions and is measured with the following questions: ‘Do you think the current health care resources are adequate?”, “Is the service resources’ distribution across different regions balanced?”, “Is it serious that public health services are market-oriented and insufficiently public?”, and “Is it convenient to get public health services?”. Answers were chosen from a 5-point scale: adequacy of medical service resources (from most adequate to least adequate), Balance of service resources’ distribution (from most balanced to most uneven), market-oriented and insufficiently public of public health services (from most serious to not serious at all), and convenience of access to public health services (from most convenient to not convenient at all).

Referring to related research, in our analysis, each dimension was dichotomized into two groups. For example, market-oriented and insufficiently public of public health services was dichotomized as sufficiently public (when the answer was not too serious, not serious at all) and insufficiently public (when the answer was most serious, more serious or neutrality). Adequacy of medical service resources was dichotomized as adequate (when the answer was most adequate or more adequate) and inadequate (when the answer was least adequate, less adequate or neutrality), and so on for other dimensions; respectively, balanced and unbalanced, and convenient and inconvenient. For details, please refer to each table.

***Independent variables.*** The related studies differ in their choice of independent variables. Factors such as sex, age, health status, and household economic status were used as independent variables in a study of residents’ satisfaction with the health care system in the former Soviet Union countries [25]. The US scholars incorporated health status, and personal annual income, into their study of people’s satisfaction with health care systems in 21 countries [26]. In the study of people’s satisfaction and perceived equality of medical service in China from 2006 to 2019, Chinese scholars used hukou, perceived social equality, and household income as the independent variables [14]. Socioeconomic status was used as a crucial independent variable in a survey on Iran residents’ satisfaction with the health care system [27].

Based on existing studies and with reference to the Anderson’s behavioral model, this study selected sex, age, marital status, education level, job, personal annual income, household income, medical insurance participation, pension, health status, perceived social trust, perceived social equity, and perceived social class as independent variables. In addition, the economic regional division was included as an independent variable innovatively.

***Sociodemographic factors.*** Specifically, the Sociodemographic factors included sex, age, hukou (household registration), and education level. These were selected for the analysis based on prior literature on the satisfaction of public health services. 

***Hukou.*** In China, the hukou system is a mandatory household registration system. Every Chinese resident is assigned a type of household registration shortly after birth, which entitles them to certain social benefits [28]. The hukou system includes rural and non-rural hukou. It has different effects on residents’ access to education, employment, healthcare, and other social benefits [29].

***Economic zone.*** There are regional imbalances in China’s economic development. Now China is divided into 4 economic zones by the National Bureau of Statistics, respectively, the eastern (10 provinces), middle (6 provinces), western (12 provinces), and northeastern zones (3 provinces have recently established as the northeastern zone). 

Additional content on how these variables were categorized is available in the tables.

### 2.3. Statistical Analysis

In this study, a chi-square test was used to conduct a univariate analysis of whether there were statistical differences between residents’ evaluation of medical resource allocation and their socio-demographic factors. In addition, multivariate logistic regression models were used to test the relationship between residents’ socio-demographic factors and their evaluation of medical resource allocation. The Odds Ratio (OR) and its 95% confidence interval (95%CI) were calculated. The basic model is as follows:

$$ogit\left(\pi \right)=ln\left(\frac{\pi }{1-\pi }\right)=\ln\left(Odds\right)=\beta_{0}+\beta_{1}X_{1}+\cdots +\beta_{p}X_{p}$$

When the probability of an event (e.g., people think service resources are adequate) occurring is π, then 1−π is probability of that event not occurring. The odds of that event are the ratio between the two probabilities. β_0_ is a constant term. β_1_…β_p_ are regression coefficients. X_1_…X_p_ are the independent variables.

When X_j_ increases by n units, ln (Odds) changes:

$$\ln\left({Odds}_{0}\right)=\beta_{0}+\beta_{1}X_{1}+\cdots {+\beta }_{j}X_{j}+\cdots +\beta_{p}X_{p}$$

$$\ln\left({Odds}_{1}\right)=\beta_{0}+\beta_{1}X_{1}+\cdots {+\beta }_{j}{(X}_{j}+n)+\cdots +\beta_{p}X_{p}$$

$$ln\left(\frac{{Odds}_{1}}{{Odds}_{0}}\right)=ln\left({Odds}_{1}\right)-ln\left({Odds}_{0}\right)=n\beta_{j}$$

Therefore, we got OR as follows:

$$OR=\frac{{Odds}_{1}}{{Odds}_{0}}=e^{n\beta_{j}}$$

All data in this paper were analyzed using SPSS software, and all statistical tests were two-sided, with *p*-values of <0.05 (*p* < 0.05) considered statistically significant.

## 3. Results

A total of 11,438 samples were obtained from the CGSS2013 questionnaire, which was screened according to the study’s research objectives. Finally, 5360 residents responded to the question, “Do you think the current health care resources are adequate?”, 5326 residents responded to the question, “Is the service resources’ distribution across different regions balanced?”, 5228 residents responded to the question “Is it serious that public health services are market-oriented and insufficiently public?”, 5393 residents answered, “Is it convenient to get public health services?”

### 3.1. Univariate Analysis

As indicated in Table 1, residents’ evaluation of the adequacy of medical resource allocation increased with age (*p* < 0.001) and was influenced by marital status, education level, job, household income, health status, pension, perceived social trust, perceived social equity, perceived social class and economic zone (*p* < 0.05). More than half of residents who had the highest evaluation of social equity (chose “totally fair”) thought that medical resources are adequate, accounting for 57.86%.

Residents’ evaluation of balance had statistical differences with their age, education level, job, perceived health status, perceived social trust, perceived social equity, perceived social class, and economic zone (*p* < 0.05). Moreover, the higher the residents’ evaluation of social trust and social equity, the higher their evaluation of the balance of medical resource allocation. In addition, the higher the level of education, the lower the residents’ evaluation of balance. 

Significant differences were found in the evaluation of publicness between the influences of education level, hukou, job, perceived health status, medical insurance, pension, perceived social trust, perceived social equity, and economic zone (*p* < 0.05).

Residents’ evaluation of accessibility differed from age, marital status, household income, perceived health status, pension, perceived social trust, perceived social equity, perceived social class, and economic zone, with statistical difference (*p* < 0.05). Residents with higher household incomes and perceived social status agreed that access to health care public services was convenient.

It is noteworthy that there were significant differences between the evaluations of all four dimensions and residents’ economic zone. In addition, residents in the central region had a lower evaluation of adequacy, balance, and publicness than residents in the other three economic zones.

### 3.2. Logistic Regression Analysis

Table 2 shows the results of the binary logistic regression. In terms of the evaluation of the adequacy of resources for public health services, residents aged 31–50 or over 70 years had higher evaluations of medical resource adequacy than residents under 30 years old (OR = 1.339, 95%CI: 1.009 to 1.776; OR = 3.675, 95%CI: 1.320 to 10.235). Those residents who perceived their social class to be in the upper middle class were more likely to agree that medical resources were adequate than those who perceived themselves to be in the lower class (OR > 1). Compared to the residents in the eastern region, central residents had lower evaluations (OR = 0.697, 95% CI: 0.529~0.918).

From the perspective of the balance of medical resource allocation, female residents had a higher evaluation than male residents. Residents who perceived social trust to be neutral and agreeable, who perceived social equity to be fair and totally fair, and who perceived higher social class were more likely to agree that the distribution of medical resources was balanced (OR > 1). Residents in the central region were less likely to agree that the distribution of medical resources was balanced than those in the eastern region (OR = 0.642, 95% CI: 0.464–0.889).

Regarding the evaluation of the publicness of medical resource allocation, compared with the reference group (residents who perceived social equity as completely unfair), residents who perceived social equity as fair or totally fair perceived less serious insufficiencies in the publicness of public health services (OR = 2.140, 95% CI: 1.133 to 4.043; OR = 3.130, 95% CI: 1.192 to 8.223). In addition, the results showed that females, employees of enterprises, residents who participated in medical insurance, and those in the middle class were the contributing factors in recognizing the publicness of medical resources (OR > 1).

In terms of accessibility of medical resource allocation, those who agreed or strongly agreed with social trust and those who perceived the social equity as totally fair were more likely to have a higher evaluation of the accessibility of medical services (OR > 1).

### 3.3. Dimensions Comparison

Residents’ evaluation of different dimensions of medical resources varied widely. A total of 49.66% of residents thought that access to public health services was convenient. Secondly, 37.95% of residents thought that public health services resources were adequate, and 21.00% of residents thought that the distribution of public health services resources was balanced across different regions. The least number of residents, accounting for only 14.46%, thought market-oriented public health services and insufficiently public were not serious.

A comparison of the four binary logistic regression models revealed that there were also similarities and differences in the influencing factors that differed significantly from the four dimensions. Firstly, the variables in the Anderson’s behavioral model, including sex, age, job, and medical insurance participation, differed significantly from residents’ evaluations of the adequacy, balance, and publicness of medical resources but did not varied from the accessibility of medical resources. Secondly, the four dimensions had closer relationships with variables such as perceived social trust, perceived social equity, perceived social class, and economic zone than those in the Anderson’s behavioral model, and some factors were used as influencing factors in multiple dimensions at the same time. For example, there were significant differences between perceived social trust and the evaluation of balance and accessibility. The economic zone in the central region was a hindering factor for residents in evaluating the adequacy and balance of medical resources. There were significant differences between perceived social equity and the evaluation of balance, publicness, and accessibility. Perceived social class was closely related to adequacy, balance, and publicness.

## 4. Discussion

In this study, we divided the evaluation of medical resource allocation into four dimensions. Residents differed in their agreement on these four dimensions, selecting them from highest to lowest were accessibility of medical resources, adequacy of medical resources, balance of medical resources allocation, and publicness of medical resources. 

The results showed that residents’ evaluation of the accessibility of medical resources was the highest in these four evaluation dimensions, which may benefit from China’s commitment of improving the network of the primary medical institutions, enhancing the service capacity of the primary medical and health institutions, and implementing the coverage of basic medicine systems in the primary medical institution [30]. Furthermore, perceived social trust and perceived social equality were the promoting factors in the accessibility evaluation of medical resource allocation, while medical insurance participation was not significantly different. The result was different from the study in the United States, which found that medical insurance premium was a hindering factor in residents’ evaluation of the accessibility of medical resources [18]. The reasons for this difference may lie in the following points. First, the medical insurance system is different. In the American medical insurance system, commercial medical insurance is dominant. High premiums and the cost of medical treatment increase the burden of health care on residents and reduce the accessibility of medical resources for the poor and elderly [31]. Second, there are differences in medical insurance coverage. The United States is the only developed country that has not achieved universal medical insurance coverage, while China’s basic medical insurance coverage will reach over 95% by 2021 [32].

In our study, higher evaluations of medical resource adequacy were found among residents aged 31–50 or over 70 years, not among those aged 51–69 years. This finding was similar to that of our previous study [33]. There might be two hypothetical reasons. First, compared with residents aged over 75 years, those middle-aged residents were more likely to report “not see a doctor when they are ill” [34]. In other words, these middle-aged people use medical services less frequently than older people. Lower usage may result in lower satisfaction with the medical system. Second, younger age groups usually use mobile medical services more often than middle-aged people. The pleasant experience of fast medical services made the younger group evaluate medical resources more highly.

We also found that residents who perceived their social class to be in the upper middle class were more likely to agree that medical resources were adequate and balanced. People from the upper middle class may have more social resources, including medical resources. With these social and medical resources, they may get better health services in most cases. Therefore, they were generally satisfied with the current allocation of medical resources. Moreover, female residents had a higher evaluation of the balance of medical resource allocation in China. In many social fields, such as medical care, Chinese society has cultural traditions that care for women and children in particular. For example, there are special hospitals for women and children in many areas of China, such as the Maternal and Child Health Hospital. Therefore, in the case of China, women may be more satisfied with the medical balance from a macro perspective.

Regional economic differences influence residents’ evaluation of the adequacy and balance of medical resource allocation. The results of this study showed that residents in the central region had a lower evaluation of the adequacy of medical resources and the balance of resource distribution than residents in the eastern region. This result may be due to the higher economic level and abundant medical resources in the eastern region of China. Moreover, the Chinese government has implemented the Strategy for Large-scale Development of Western China since 2000, which has tilted the fiscal investment in medical resources in the western region [35]. Although the overall medical resources in the eastern region are more abundant, due to the difference in the economic development level within the eastern region, the inequity of medical resource allocation is more severe than that in the central and western regions [2]. Therefore, it is necessary to pay attention not only to the imbalanced development among the four economic zones but also to the impact of economic development within each economic zone on the allocation of medical resources.

A total of 85.54% of residents agreed that public medical and health services were too market-oriented and lacked publicness. First of all, those who perceived a higher degree of social equity and those who perceived their social class to be middle class thought that the lack of publicness was not serious. The role of the market mechanism in improving the efficiency of medical services cannot be ignored, but at the same time, marketization may intensify medical institutions’ profit-pursing behavior, causing high-quality medical resources to concentrate in economically developed regions and higher income groups, resulting in significant differences in medical resources between regions with different levels of economic development and between urban and rural areas. China’s new round of medical and health system reform has changed the situation of excessive marketization of medical services to some extent, but hospitals are still able to profit from inducing patients’ medical needs and other means [36]. Secondly, the residents who participated in medical insurance thought that the lack of publicness was not serious, that is, the medical insurance coverage is a positive contributor to the publicness of health care services. The US, for example, is a representative country with a high degree of marketization of health care services, relying mainly on market mechanisms to solve the problems of health care services. Moreover, it is dominated by commercial medical insurance, so that medical insurance does not cover the entire population, and its basic health services have difficulties in meeting the health needs of vulnerable groups [37]. Studies found that the expansion of medical insurance coverage in Mexico has reduced catastrophic expenditures for the population, especially for the poor [38]. Although marketization brings high efficiency, it will lead to a lack of publicness and equality in medical and health services.

Previous studies on residents’ evaluation of medical resource allocation generally evaluated residents’ satisfaction with medical resource allocation, whereas this study divided satisfaction into four dimensions and analyzed the results from multiple perspectives to make the results more pertinent. Furthermore, the four dimensions were compared in the same study to provide a clearer picture of the factors that influence residents’ evaluation of medical resources allocation, as well as their specific demands for medical resources, and provide a reference for policy formulation and practice.

However, there are still some limitations in this study. This study used CGSS data from 2013, and the analysis results were more consistent with residents’ evaluation of medical resource allocation at that time, which may be different from the current situation to some extent. The data from CGSS2013 were selected mainly because only the data from 2013 in the CGSS database contain the four dimensions of data of medical resource evaluation used in this study, which we still believe has a certain reference value.

## 5. Conclusions

This study used data from CGSS2013 to subdivide the dimensions of evaluation of medical resource allocation into four dimensions, including adequacy, balance, publicness, and accessibility, and to explore residents’ evaluation of these four dimensions of medical resource allocation and their influencing factors. The findings show that residents’ agreement on the publicness of medical resource allocation is low, only 14.46%, while their agreement on accessibility is high, nearly 50%. The study also finds that sex, age, perceived social trust, perceived social equity, perceived social class, and economic regional division all had an impact on residents’ evaluation of medical resource allocation.

This study enriches the theoretical research on the evaluation of medical resource allocation. Firstly, in terms of the dimensions of the study, this study is enriched and refined compared to previous studies so that the dimensions of evaluation of medical resource allocation are subdivided and supplemented from the single equity and accessibility to four dimensions, including adequacy, balance, publicness, and accessibility. Secondly, a comparative study of the above dimensions is conducted to analyze further the similarities and differences in residents’ evaluation of these dimensions. Finally, in terms of the choice of influencing factors, in addition to sex, job, education level, perceived social trust, and perceived social equity, economic regional division, and the hukou system with Chinese characteristics are added.

This study may provide a reference for the practice of medical resource allocation in China. The findings of the study provide residents’ evaluation of multiple dimensions of medical resource allocation and a comparative study between the dimensions, which helps to improve the relevance and precision of medical resource allocation policy formulation. The following three recommendations are made based on the findings of this study: Firstly, to enhance the publicness of public health services, it is important to promote equal access to public service to increase residents’ evaluation of social equity. The government should play a proper role in supervising and managing the market for health services and maintain the public welfare of health services. Secondly, increase financial support for the central region, especially increasing investment in the health field, and reducing the differences in economic development levels between economic zones and within economic zones. Thirdly, set up different medical resource allocation schemes according to social class differences and establish a multi-level medical security system.

## Figures and Tables

**Table 1:** Bivariate Associations Between Sociodemographic Characteristics and Service resource evaluation among CGSS (2013) Respondents.

	Adequacy of Service Resources Provided by Public Health Services (N = 5360)	Balance of Service Resources’ Distribution across Different Regions (N = 5252)	Is it Serious that Public Health Services Are Market-oriented and Insufficiently Public? (N = 5228)	Convenience of Access to Public Health Services (N = 5393)
	***Adequant*** ***(N = 2034, 37.95%)***	***Balanced*** ***(N = 1103, 21.00%)***	***Not serious*** ***(N = 756, 14.46%)***	***Convenient*** ***(N = 2678, 49.66%)***
**Sex**	*p* ^1^ = 0.508	*p* = 0.192	*p* = 0.634	*p* = 0.864
Male	37.51% (1017)	20.28% (540)	14.23% (377)	49.54% (1352)
Female	38.39% (1017)	21.75% (563)	14.7% (379)	49.77% (1326)
**Age**	*p* < 0.001 ***	*p* = 0.044 *	*p* = 0.007	*p* = 0.019 *
≤30	30.26% (259)	18.04% (153)	12.59% (106)	45.09% (386)
31–50	37.09% (773)	22% (450)	15.31% (314)	49.62% (1040)
51–69	40.13% (716)	20.44% (357)	13.13% (228)	51% (917)
≥70	44.97% (286)	23.37% (143)	18.03% (108)	52.1% (335)
**Marriage**	*p* = 0.022 *	*p* = 0.203	*p* = 0.273	*p* = 0.011 *
Married	38.74% (1610)	21.4% (872)	14.74% (598)	50.56% (2114)
Others ^2^	35.1% (418)	19.67% (229)	13.46% (156)	46.37% (556)
**Education level**	*p* < 0.001 ***	*p* = 0.03 *	*p* = 0.016 *	*p* = 0.064
Eementary school level or below	42.89% (811)	22.61% (412)	16.26% (291)	50.58% (963)
Junior/senior middle school or technical secondary school	36.22% (940)	20.78% (532)	13.9% (357)	50.21% (1313)
Undergraduate or above	32.42% (283)	18.3% (159)	12.43% (108)	46.05% (402)
**Hukou**	*p* = 0.650	*p* = 0.735	*p* = 0.006 **	*p* = 0.895
Rural resident	38.24% (1136)	21.17% (613)	15.7% (449)	49.65% (1491)
Non-rural resident	37.63% (896)	20.78% (488)	13.01% (307)	49.83% (1187)
**Job**	*p* = 0.002 **	*p* = 0.011 *	*p* = 0.023 *	*p* = 0.102
Government department or institution	34.72% (150)	19.72% (85)	16.44% (71)	47.34% (205)
Enterprise	33.05% (315)	21.04% (199)	11.39% (108)	49.06% (469)
Self-employed or others	41.12% (322)	26.2% (202)	14.56% (113)	53.11% (419)
**Personal annual income (RMB)**	*p* = 0.874	*p* = 0.155	*p* = 0.246	*p* = 0.523
Low (<10,000)	39.18% (652)	20.3% (327)	16.11% (255)	49.76% (839)
Median (10,000~29,999)	38.46% (633)	23.1% (373)	14.9% (242)	51.72% (855)
High (≥30,000)	39.27% (589)	21.62% (321)	13.96% (208)	50.9% (764)
**Perceived household income**	*p* < 0.001 ***	*p* = 0.107	*p* = 0.148	*p* = 0.006 **
Below average	34.95% (626)	19.47% (341)	14.7% (255)	46.98% (849)
Average	38.82% (1217)	21.54% (663)	13.97% (429)	50.63% (1596)
Above average	44.31% (183)	23.46% (95)	17.57% (71)	54.48% (225)
**Health Status**	*p* < 0.001 ***	*p* < 0.001 ***	*p* < 0.001 ***	*p* = 0.001 **
Very bad	43.14% (66)	15.97% (23)	26.95% (38)	50.32% (78)
Bad	39.39% (282)	19.1% (131)	16.2% (111)	47.51% (344)
Average	32.07% (322)	16.92% (167)	10.47% (103)	45.67% (464)
Healthy	38.41% (802)	21.62% (445)	14.6% (298)	49.45% (1037)
Very healthy	40.2% (562)	24.49% (337)	14.97% (206)	53.86% (754)
**Access to medical insurance ^3^ (not include commercial medical insurance)**	*p* = 0.151	*p* = 0.394	*p* = 0.002 **	*p* = 0.084
No	35.16% (192)	22.45% (121)	9.94% (53)	46.25% (253)
Yes	38.31% (1832)	20.87% (977)	14.97% (698)	50.15% (2414)
**Access to pension ^4^ (not include commercial pension)**	*p* = 0.001 **	*p* = 0.278	*p* = 0.028 *	*p* = 0.001 **
No	34.81% (573)	20.2% (326)	12.8% (205)	46.46% (768)
Yes	39.5% (1425)	21.53% (761)	15.13% (533)	51.35% (1865)
**Perceived social trust (the majority of people are worthy of trust)**	*p* < 0.001 ***	*p* < 0.001 ***	*p* < 0.001 ***	*p* < 0.001 ***
Level 1 (strongly disagree)	34.83% (93)	16.99% (44)	12.74% (33)	45.69% (122)
Level 2 (disagree)	33.49% (420)	17.3% (213)	12.99% (160)	42.79% (540)
Level 3 (neutrality)	33.05% (269)	19% (152)	11.98% (95)	43.59% (357)
Level 4 (agree)	40.7% (1114)	23.04% (618)	15.16% (405)	53.96% (1484)
Level 5 (strongly agree)	48.24% (137)	27.54% (76)	22.88% (62)	59.66% (173)
**Perceived social equity**	*p* < 0.001 ***	*p* < 0.001 ***	*p* < 0.001 ***	*p* < 0.001 ***
Level 1 (totally unfair)	28.68% (109)	14.32% (53)	8.92% (33)	41.41% (159)
Level 2 (unfair)	31.76% (491)	14.37% (219)	11.23% (171)	45.78% (711)
Level 3 (neutrality)	33.83% (432)	19.1% (237)	12.13% (151)	45.65% (588)
Level 4 (fair)	45.55% (907)	27.07% (529)	18.53% (358)	55.68% (1113)
Level 5 (totally fair)	57.86% (92)	41.03% (64)	27.63% (42)	63.58% (103)
**Perceived social class (Which level do you think you are in the social class?)**	*p* < 0.001 ***	*p* < 0.001 ***	*p* = 0.792	*p* = 0.006 **
Bottom	33.69% (529)	16.56% (253)	13.93% (211)	46.62% (738)
Middle	38.8% (1305)	22.15% (733)	14.58% (481)	50.44% (1705)
Upper	46.45% (190)	27.82% (111)	14.96% (60)	54.26% (223)
**Regional division based on economic level**	*p* < 0.001 ***	*p* < 0.001 ***	*p* < 0.001 ***	*p* = 0.031 *
East region	39.99% (789)	23.01% (445)	13.5% (261)	50.2% (993)
Central region	32.13% (428)	15.41% (199)	9.91% (127)	47.63% (643)
West region	41.64% (558)	24.14% (317)	20.09% (261)	52.41% (707)
North-east region	36.22% (259)	19.89% (142)	14.99% (107)	46.79% (335)

^1^ *p* for Chi-square test. * *p* < 0.05, ** *p* < 0.01, *** *p* < 0.001. ^2^ include unmarried, cohabitation, separated and not divorced, divorce and widowed. ^3^ include urban medical insurance, new cooperative medical insurance, and public medical insurance. ^4^ include rural pension, urban residents’ pension.

**Table 2:** Binary Logistic Regression Model of Respondents’ Sociodemographic Characteristics and Service resource evaluation among CGSS (2013).

	Adequacy of Service Resources Provided by Public Health Services *(Adequant: 37.95%)*		Balance of Service Resources’ Distribution across Different Regions *(Balanced: 21.00%)*		Is it Serious that Public Health Services Are Market-Oriented and Insufficiently Public? *(Not Serious: 14.46%)*		Convenience of Access to Public Health Services *(Convenient: 49.66%)*	
	**OR (95%CI)**	***p* ^1^**	**OR (95%CI)**	***p* ^1^**	**OR (95%CI)**	***p* ^1^**	**OR (95%CI)**	***p* ^1^**
**Sex**								
Male	Reference		Reference		Reference		Reference	
Female	1.145 (0.935–1.401)	0.190	1.393 (1.104–1.758)	0.005 **	1.332 (1.011–1.754)	0.041 *	1.122 (0.923–1.363)	0.248
**Age**								
≤30	Reference		Reference		Reference		Reference	
31–50	1.339 (1.009–1.776)	0.043 *	1.182 (0.852–1.640)	0.317	1.292 (0.872–1.914)	0.202	1.016 (0.777–1.329)	0.909
51–69	1.433 (0.999–2.054)	0.050	1.007 (0.660–1.534)	0.976	1.054 (0.628–1.768)	0.843	1.033 (0.731–1.458)	0.855
≥70	3.675 (1.320–10.235)	0.013 *	1.717 (0.586–5.032)	0.324	2.189 (0.676–7.089)	0.191	1.169 (0.434–3.148)	0.757
**Marriage**								
Married	Reference		Reference		Reference		Reference	
Others ^2^	0.957 (0.724–1.264)	0.757	0.850 (0.611–1.184)	0.337	1.109 (0.756–1.628)	0.596	0.820 (0.629–1.069)	0.143
**Education level**								
Eementary school level or below	Reference		Reference		Reference		Reference	
Junior/senior middle school or technical secondary school	1.011 (0.739–1.385)	0.944	0.873 (0.611–1.248)	0.457	1.370 (0.869–2.161)	0.176	0.989 (0.727–1.347)	0.946
Undergraduate or above	0.852 (0.569–1.275)	0.436	0.684 (0.430–1.089)	0.110	1.248 (0.698–2.228)	0.455	0.866 (0.585–1.281)	0.471
**Hukou**								
Rural resident	Reference		Reference		Reference		Reference	
Non-rural resident	0.863 (0.688–1.084)	0.205	0.916 (0.705–1.190)	0.510	0.741 (0.540–1.016)	0.063	0.969 (0.778–1.208)	0.780
**Job**								
Government department or institution	Reference		Reference		Reference		Reference	
Enterprise	0.886 (0.674–1.164)	0.384	0.995 (0.719–1.378)	0.977	0.626 (0.434–0.902)	0.012 *	1.033 (0.795–1.341)	0.808
Self-employed or others	1.251 (0.928–1.687)	0.142	1.406 (0.990–1.996)	0.057	0.833 (0.561–1.236)	0.364	1.192 (0.892–1.594)	0.235
**Personal annual income (RMB)**								
Low (<10,000)	Reference		Reference		Reference		Reference	
Median (10,000~29,999)	0.995 (0.682–1.453)	0.980	1.164 (0.748–1.814)	0.501	0.979 (0.586–1.636)	0.936	0.860 (0.598–1.238)	0.418
High (≥30,000)	0.999 (0.670–1.491)	0.998	1.072 (0.669–1.718)	0.773	0.864 (0.501–1.491)	0.600	0.802 (0.546–1.177)	0.259
**Perceived household income**								
Below average	Reference		Reference		Reference		Reference	
Average	1.095 (0.861–1.393)	0.459	0.874 (0.662–1.154)	0.344	0.961 (0.687–1.345)	0.818	1.192 (0.947–1.500)	0.135
Above average	1.122 (0.760–1.655)	0.563	0.732 (0.463–1.155)	0.180	1.118 (0.661–1.893)	0.677	1.194 (0.816–1.746)	0.362
**Health Status**								
Very bad	Reference		Reference		Reference		Reference	
Bad	1.538 (0.402–5.883)	0.529	1.838 (0.200–16.889)	0.591	0.687 (0.123–3.848)	0.670	1.235 (0.347–4.396)	0.744
Average	1.022 (0.275–3.797)	0.974	2.554 (0.289–22.527)	0.399	0.398 (0.073–2.180)	0.289	0.963 (0.280–3.316)	0.953
Healthy	1.354 (0.369–4.974)	0.648	2.619 (0.299–22.933)	0.384	0.584 (0.110–3.115)	0.529	1.169 (0.343–3.990)	0.803
Very healthy	1.796 (0.488–6.608)	0.378	3.699 (0.422–32.394)	0.238	0.624 (0.117–3.336)	0.582	1.543 (0.451–5.279)	0.489
**Access to medical insurance ^3^ (not include commercial medical insurance)**								
No	Reference		Reference		Reference		Reference	
Yes	1.026 (0.741–1.419)	0.878	0.834 (0.578–1.205)	0.334	1.817 (1.082–3.050)	0.024 *	1.108 (0.810–1.514)	0.522
**Access to pension ^4^ (not include commercial pension)**								
No	Reference		Reference		Reference		Reference	
Yes	1.052 (0.827–1.337)	0.680	1.125 (0.851–1.488)	0.409	1.019 (0.729–1.422)	0.914	1.085 (0.861–1.367)	0.490
**Perceived social trust (the majority of people are worthy of trust)**								
Level 1 (strongly disagree)	Reference		Reference		Reference		Reference	
Level 2 (disagree)	0.759 (0.489–1.178)	0.219	1.534 (0.820–2.869)	0.181	1.184 (0.601–2.330)	0.625	0.908 (0.593–1.390)	0.657
Level 3 (neutrality)	0.744 (0.464–1.193)	0.220	1.942 (1.014–3.717)	0.045 *	1.081 (0.526–2.222)	0.832	0.965 (0.612–1.522)	0.879
Level 4 (agree)	0.956 (0.625–1.462)	0.836	2.198 (1.200–4.028)	0.011 *	1.254 (0.650–2.417)	0.499	1.591 (1.053–2.405)	0.028 *
Level 5 (strongly agree)	1.220 (0.673–2.214)	0.512	2.091 (0.964–4.535)	0.062	1.013 (0.415–2.477)	0.977	1.841 (1.020–3.322)	0.043 *
**Perceived social equity**								
Level 1 (totally unfair)	Reference		Reference		Reference		Reference	
Level 2 (unfair)	0.847 (0.581–1.235)	0.388	0.750 (0.465–1.211)	0.240	1.482 (0.785–2.797)	0.225	0.999 (0.697–1.432)	0.997
Level 3 (neutrality)	0.814 (0.548–1.209)	0.308	1.081 (0.665–1.757)	0.754	1.780 (0.927–3.416)	0.083	0.908 (0.623–1.325)	0.617
Level 4 (fair)	1.170 (0.796–1.718)	0.425	1.666 (1.042–2.663)	0.033 *	2.140 (1.133–4.043)	0.019 *	1.314 (0.907–1.904)	0.149
Level 5 (totally fair)	1.756 (0.846–3.647)	0.131	2.302 (1.046–5.069)	0.038 *	3.130 (1.192–8.223)	0.021 *	2.240 (1.027–4.886)	0.043 *
**Perceived social class (Which level do you think you are in the social class?)**								
Bottom	Reference		Reference		Reference		Reference	
Middle	1.312 (1.015–1.696)	0.038 *	1.774 (1.285–2.449)	0.001 **	1.550 (1.057–2.272)	0.025 *	0.906 (0.712–1.153)	0.421
Upper	1.792 (1.211–2.651)	0.004 **	2.120 (1.336–3.364)	0.001 **	1.376 (0.783–2.419)	0.268	0.994 (0.678–1.457)	0.976
**Regional division based on economic level**								
East region	Reference		Reference		Reference		Reference	
Central region	0.697 (0.529–0.918)	0.010 *	0.642 (0.464–0.889)	0.008 **	0.671 (0.450–1.001)	0.051	0.777 (0.599–1.009)	0.058
West region	0.873 (0.669–1.139)	0.317	0.810 (0.594–1.104)	0.182	1.205 (0.847–1.714)	0.301	0.924 (0.714–1.195)	0.545
North-east region	0.893 (0.653–1.222)	0.479	0.778 (0.537–1.128)	0.185	0.990 (0.639–1.533)	0.963	0.896 (0.662–1.213)	0.477

^1^ *p* for Logistic Regression model. * *p* < 0.05, ** *p* < 0.01. ^2^ include unmarried, cohabitation, separated and not divorced, divorce and widowed. ^3^ include urban medical insurance, new cooperative medical insurance, and public medical insurance. ^4^ include rural pension, urban residents’ pension.

## Data Availability

The datasets CGSS2013 for this study is a public database, and can be found on the website http://www.cnsda.org/index.php?r=projects/view&id=93281139 (accessed on 15 June 2022).
